# A histopathologic study of fatal paediatric cerebral malaria caused by mixed *Plasmodium falciparum*/*Plasmodium vivax *infections

**DOI:** 10.1186/1475-2875-11-107

**Published:** 2012-04-03

**Authors:** Laurens Manning, Anna Rosanas-Urgell, Moses Laman, Henry Edoni, Catriona McLean, Ivo Mueller, Peter Siba, Timothy ME Davis

**Affiliations:** 1 School of Medicine and Pharmacology, University of Western Australia, Fremantle Hospital, PO Box 480, Fremantle 6959, Western Australia, Australia; 2 Papua New Guinea Institute of Medical Research, Madang, Papua New Guinea; 3 Department of Medicine, Faculty of Medicine Nursing and Health Sciences, Alfred Hospital, Prahran, Victoria, Australia; 4 Infection and Immunity Division, Walter and Eliza Hall Institute, Parkville, Victoria, Australia; 5 Centre de Recerca en Salut Internacional de Barcelona (CRESIB), Barcelona, Spain

**Keywords:** *Plasmodium vivax*, Post mortem biopsy, PCR, Cerebral malaria, Children

## Abstract

Microvascular sequestration of *Plasmodium falciparum *underlies cerebral malaria. Despite suggestive *ex vivo *evidence, this phenomenon has not been convincingly demonstrated in coma complicating *Plasmodium vivax *malaria. Severely-ill Papua New Guinean children with mixed *P. falciparum/P. vivax *infections are more likely to develop cerebral malaria and die than those with *P. falciparum *alone, possibly reflecting *P. vivax *sequestration. Nested PCR was performed on *post mortem *brain tissue from three such children dying from cerebral malaria due to mixed-species infections. No *P. vivax *DNA was detected. These findings do not support the hypothesis that *P. vivax *sequestration occurs in human brain.

## Background

*Plasmodium vivax *accounts for nearly half of all malaria infections and is now recognized as a cause of complications and death [1]. Cerebral malaria due to *P. vivax*, although rare, has been reported from Indonesia, India and Papua New Guinea (PNG) [2-4]. In patients with altered consciousness due to *Plasmodium falciparum*, late stage parasites (trophozoites and schizonts) can be found sequestered within the brain microvasculature. There is no convincing evidence of a similar phenomenon in *post mortem *brain specimens from patients with *P. vivax*, but available data are few and from studies in which interpretation of the histopathologic features is confounded by issues such as the possibility of unrecognized mixed-species infections [5].

Animal and *in vivo *functional studies reveal that *P. vivax *sequesters preferentially in the pulmonary and splenic microvasculature [5], consistent with clinical studies in which pulmonary manifestations and severe anaemia occur relatively frequently [2,6]. *Ex vivo *studies have shown cytoadherence of *P. vivax *to chondroitin sulphate A, erythrocyte rosetting and endothelial adherence [5,7,8], albeit at lower avidity than *P. falciparum*. Nevertheless, these observations raise the possibility that cerebral microvascular sequestration may underlie cerebral malaria due to *P. vivax*.

Apart from research settings [9], autopsy studies of patients dying from malaria are rarely performed. In the case of fatal cerebral vivax malaria, there have been no published *post mortem *studies for > 60 years [5]. The results of a prospective observational study of severely ill PNG children with malaria were reported recently [2]. Those with mixed *P. falciparum/P. vivax *infections were more likely to have impaired consciousness and to die than those infected with *P. falciparum *alone. In the present study, histopathologic and molecular studies were performed using brain tissue from three of the fatal cases in this series [2] in order to examine the hypothesis that cerebral sequestration of *P. vivax *contributed to their presenting neurological features and outcome.

Details of the main prospective observational study from which the present cases are drawn have been published [2]. In brief, all children aged 0.5-10 years admitted to Modilon Hospital, Madang Province, between October 2006 and December 2009 were assessed for recruitment to an observational study of severe paediatric illness. Inclusion criteria reflected the World Health Organization (WHO) definition of severe malarial illness. All children were screened for malaria by both blood film microscopy and subsequent diagnostic and *Plasmodium *species confirmation by nested polymerase chain reaction (nPCR) [10]. In-patient management was co-ordinated by attending ward clinicians under PNG national guidelines.

Cerebral malaria was defined as a Blantyre Coma Score ≤2 with *Plasmodium *DNA detected in peripheral blood by nPCR [2], irrespective of the presence and/or species of asexual parasites by microscopy. A mixed *P. falciparum/P. vivax *infection was defined as the presence of both species DNA by nPCR, regardless of species or parasitaemia detected microscopically. *Post mortem *brain tissue was obtained using a bone marrow biopsy needle via a supra-orbital approach [9]. Small fragments of brain tissue were placed between two slides, gently squashed, stained with 2.5% Giemsa for one hour before microscopic examination. Additional pieces of brain tissue were stored in formalin and liquid nitrogen. Formalin-fixed tissue was embedded in paraffin, sectioned and stained with haematoxylin and eosin (H&E), Gram's, Giemsa and silver stain prior to microscopy which was performed by a specialist neuropathologist (CM) in a pathology laboratory accredited by the Australian National Association of Testing Authorities.

Initial *Plasmodium *species identification was performed on peripheral blood by nPCR [10] after parasite DNA extraction (QIAamp 96 DNA Blood Mini Kit, QIAGEN, Valencia, CA). Positive samples for mixed *P. falciparum*/*P. vivax *infection were quantified by PCR (qPCR) using validated methods [11]. Brain tissue stored in liquid nitrogen or formalin was homogenized before DNA extraction (DNeasy Blood & Tissue Kit, QIAGEN, Valencia, CA, USA) prior to nPCR.

## Case presentations

Three children of 340 with severe malaria died from cerebral malaria and underwent *post mortem *brain biopsy. No brain biopsy material was available for the other three children in the series as a whole who died. All three of the present children had a mixed *P. falciparum*/*P. vivax *infection detected from peripheral blood by nPCR. Their admission clinical and laboratory features are summarized in Table 1.

**Table 1:** Clinical and laboratory features of children dying from cerebral malaria due to mixed *Plasmodium falciparum/Plasmodium vivax *infections

	Patient 1	Patient 2	Patient 3
Age (months)	63	36	94

Sex	Female	Female	Male

Blantyre coma score on admission	0	2	1

Blood lactate (mmol/L)	1.6	6.4	1.0

Plasma bicarbonate (mmol/L)	18	10.2	22.3

Blood glucose (mmol/L)	18.4	3.2	7.0

Haemoglobin (g/L)	69	39	111

Peripheral parasitaemia (/μL) by light microscopy:			

*P. falciparum*	154,000	104,000	0

*P. vivax*	0	80	0

*Plasmodium *species by nPCR (blood)	Mixed species	Mixed species	Mixed species

Peripheral parasitaemia by qPCR (number of cycles):			

*P. falciparum*	26.3	23.1	37.2

*P. vivax*	41.0	29.2	35.8

*Plasmodium *species by nPCR (brain)	*P. falciparum*	*P. falciparum*	Negative

nPCR, nested polymerase chain reaction [10]; qPCR, quantitative polymerase chain reaction [11]

### Case 1

**Figure 1:**
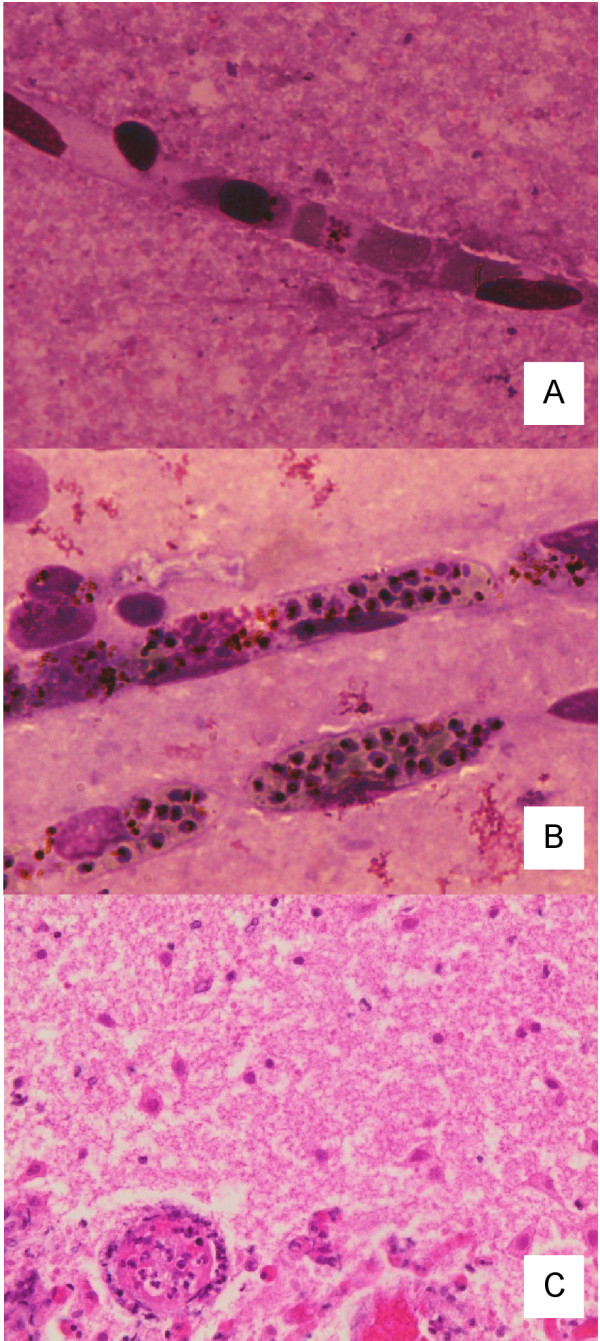
**Light microscopy of brain tissue obtained from children dying from cerebral malaria due to mixed Plasmodium falciparum/Plasmodium vivax infections**. In panel **A **(Patient 1), occasional mature forms of *P. falciparum *were seen (Giemsa, magnification × 400). In panel **B **(Patient 2), malaria pigment and numerous *P. falciparum *trophozoites and schizonts are visible within the microvasculature (Giemsa, magnification x400). In panel **C **(Patient 3), there is acute chromatolysis of neurons and early infiltration by neutrophils from adjacent vessels at the edge of an area of infarction. The cerebral blood vessel (lower left) was normal and no malaria parasites were seen (H&E, magnification × 400).

This child presented in deep coma without other features of severe malaria and died within hours of admission despite prompt anti-malarial therapy and supportive treatment. *P. falciparum *was present on blood smear and nPCR, but *P. vivax *was not identified by microscopy and only at low levels (> 40 cycles) by qPCR. The qPCR data represent a *P. falciparum *and *P. vivax *parasitaemia of 10,000 and < 10 parasites/μL, respectively [11]. Microscopy of brain tissue revealed occasional *P. falciparum *parasites and no identifiable *P. vivax *within the cerebral vasculature (Figure 1). No inflammation, haemorrhage or necrosis was observed in the adjacent brain parenchyma. *Plasmodium falciparum *but not *P. vivax *was present in brain by nPCR.

### Case 2

This patient presented with cerebral malaria, metabolic acidosis, hyperlactataemia and severe anaemia. This child died within hours of admission despite prompt anti-malarial therapy and supportive treatment. *P. falciparum *and *P. vivax *in peripheral blood were identified by microscopy, nPCR and qPCR. Using qPCR, *P. falciparum *and *P. vivax *DNA concentrations indicated equivalent parasitaemia of 50,000 and 500/μL, respectively. *Plasmodium falciparum*, but not *P. vivax*, was present in brain by nPCR. Malaria pigment and numerous *P. falciparum *trophozoites and schizonts were visible within the microvasculature on histologic examination (Figure 1). Other than subtle focal up-regulation of microglial cells, there were no abnormalities observed in the adjacent brain parenchyma.

### Case 3

This child presented deeply comatose. Seven days before admission, he attended a local clinic with headache and fever and was prescribed amodiaquine, sulphadoxine and pyrimethamine. He did not improve and was given intramuscular artemether five days later. On admission to Modilon Hospital, blood film microscopy and a rapid diagnostic test for malaria were negative, but both species were subsequently detected by nPCR of peripheral blood and at low levels by qPCR (10-100 parasites/μL). At lumbar puncture, there were 5 leucocytes/μL (100% lymphocytes) in the cerebrospinal fluid (CSF) and the CSF protein concentration was 3 g/L. Blood cultures were negative. He did not regain consciousness and died four days later despite intravenous dextrose/saline and antibiotics, and intramuscular artemether.

Neither *P. falciparum *nor *P. vivax *was present in the brain of Patient 3 by nPCR. Further molecular testing of CSF and brain tissue for arbo-, Henipah, Entero- and Herpes viruses did not reveal an alternative diagnosis. Histopathologic examination of brain tissue showed infarction of cortical tissue with acute chromatolysis of neurons and early infiltration by neutrophils from adjacent vessels at the edge of the infarction (Figure 1). Adjacent white matter was unaffected and cerebral vessels appeared normal. No lymphocytes, histiocytes, granulomata, malarial parasites, fungal hyphae, bacteria or emboli were seen. The changes were interpreted as cortical infarction of at least 24 hours duration without a definitive aetiology.

## Conclusions

The present case series provides the first detailed histopathologic and molecular study of the potential role of *P. vivax *in the pathogenesis of coma as a manifestation of severe malaria. There was no evidence for cerebral sequestration of *P. vivax *in any of the three cases despite microscopic and/or molecular confirmation that this parasite was present in peripheral blood at presentation. Thus, despite *ex vivo *studies suggesting that cytoadherence of *P. vivax *might contribute to complications [5,7,8], the present data do not support the hypothesis that this phenomenon occurs in the human brain.

Patient 1 was diagnosed and treated as a case of cerebral malaria due to a *P. falciparum *mono-infection. By implication from the peripheral blood microscopy and qPCR data, this young girl's total *P. vivax *parasite burden was relatively low and the negative cerebral histopathology and nPCR could have reflected this. Mature *P. falciparum *forms in the brain in this patient were scarce compared to the peripheral parasitaemia (154,000/μL). This suggests that she had a synchronous infection with either maturing parasite forms starting to cytoadhere, or (less likely in view of the lack of intravascular malaria pigment) maturation and rupture of schizonts just before presentation. Whether a similar phenomenon might operate in the case of *P. vivax *is unknown. In a typical patient with *P. falciparum*, about half of the infected erythrocytes are sequestered [12]. It is possible that the dynamics of putative *P. vivax *sequestration with less avid ligand binding [7] are different to *P. falciparum *in that the majority of parasites remain in the peripheral blood. This could explain the weakly positive PCR in blood and negative PCR in the brain of this patient if *P. vivax *cytoadherence were possible.

Patient 2 provides the strongest evidence against *P. vivax *cerebral cytoadherence. This child had a mixed-species infection identifiable by peripheral blood microscopy but nPCR of brain tissue was only positive for *P. falciparum*. The difference in peripheral blood *P. vivax *density observed by qPCR compared to microscopy (500 vs 80/μL, respectively) is likely to reflect the fact that even very experienced microscopists often overlook a second infecting species in mixed infections [13]. Brain histology in this patient showed an abundance of *P. falciparum *and its greater avidity for endothelial ligands under both static and flow conditions [7] may have competitively inhibited *P. vivax *cytoadherence. However, the distribution of *P. falciparum *sequestration varies significantly between cerebral vessels in the same patient [14] and, in any case, the absence of *P. vivax *by nPCR implies that this parasite had no direct role in causing deep coma.

Patient 3 was included in the present series because of the positive peripheral blood PCR results. In studies of *P. falciparum*, mature parasite forms are no longer visible in brain biopsy specimens three days after anti-malarial treatment [14] and this patient died seven days after intramuscular artemether. It is, therefore, not surprising that brain nPCR was negative for *P. falciparum *and, even if cerebral *P. vivax *sequestration had contributed to coma, that brain nPCR was also negative for *P. vivax*. Ischemia and infarction can occur in cerebral malaria caused by *P. falciparum *in children [15]. It is likely that histologic appearances in this patient, coupled with prior anti-malarial treatment, low-grade parasitaemia detected by PCR and negative investigations for other likely pathogens, reflect the adverse and persistent neurologic effects of substantial *P. falciparum *sequestration prior to admission. The role of *P. vivax *in this process remains speculative.

There were no cases of *P. vivax *mono-infections in the present *post mortem *series. Children with pure severe *vivax *malaria in the main study had a significantly greater parasitaemia than those of *P. vivax *in the mixed infection group [2], but none of these 27 children died of cerebral malaria and only one (3.7%) presented in deep coma (Blantyre Coma Score ≤ 2; *vs *10.3% for *P. falciparum *mono-infections and 22.0% for mixed species infections [2]). This is further indirect evidence against cerebral sequestration due to *P. vivax*.

The low mortality rate in the main study [2] and cultural difficulties in obtaining *post mortem *samples in PNG meant that there was limited availability of brain tissue. Nevertheless, detailed phenotypic, laboratory and outcome data were available for each of the three children. In addition, *Plasmodium *DNA was successfully extracted from brain tissue, implying that the absence of *P. vivax *DNA was not due to laboratory factors.

The present results suggest that *P. vivax *cytoadherence in brain is not a significant pathophysiologic mechanism underlying impaired consciousness in severely ill children with mixed species malaria. Other factors that underlie the poor prognosis of mixed species infections in the present series include the possibility that enhanced cytokine and/or toxin production associated with *P. vivax *[5] amplifies the effects of *P. falciparum *sequestration on vital organ function, and even that the presence of *P. vivax *in peripheral blood facilitates cytoadhesion of *P. falciparum*. The global burden of *vivax *malaria and the increasing recognition that this parasite can have adverse effects on the human host, especially in concert with *P. falciparum*, make this an important area for further study.

## Consent

Ethical approval for the main study and present sub-study was obtained from the PNG Institute of Medical Research Institutional Review Board and the Medical Research Advisory Committee of the PNG Health Department. Parent(s)/guardian(s) provided written, informed consent before a child was recruited to the main study and were subsequently asked for permission for a limited post mortem examination, including needle aspiration biopsies, if their child died.

## Abbreviations

CSF: cerebrospinal fluid; H&E: haematoxylin and eosin; nPCR: nested polymerase chain reaction; PNG: Papua New Guinea; qPCR: quantitative PCR; WHO: World Health Organization.

## Competing interests

The authors declare that they have no competing interests.

## Authors' contributions

LM co-ordinated the main study, obtained biopsies and produced the initial draft of the paper. AR-U performed the PCR assays and edited drafts of the paper. ML assisted clinically with the main study, obtained biopsies and edited drafts of the paper. HE assisted with data collection and specimen preparation, and edited drafts of the paper. CM performed the histological examination of brain tissue and edited drafts of the paper. IM was a co-investigator on the main study and edited drafts of the paper. PS was a co-investigator on the main study and edited drafts of the paper. TMED was the principal investigator of the main study, conceived the sub-study and produced the final version of the paper. All authors read and approved the final manuscript.
